# An obscure cause of gastrointestinal bleeding: Recurrent duodenal variceal hemorrhage treated with intramuscular octreotide in the absence of portal hypertension

**DOI:** 10.1002/jgh3.12847

**Published:** 2022-12-12

**Authors:** Robert S O'Neill, William J Wang, Patrick Chan, Vincent Ho, Christine Verdon, Ian Turner, Priya Acharya

**Affiliations:** ^1^ Department of Gastroenterology Campbelltown Hospital Campbelltown New South Wales Australia; ^2^ St Vincent's Clinical School, Faculty of Medicine University of New South Wales Sydney New South Wales Australia; ^3^ Western Sydney University Campbelltown New South Wales Australia; ^4^ Townsville University Hospital Douglas Queensland Australia

**Keywords:** duodenal varices, endoscopy, haemorrhage, radiology, varices

## Abstract

Duodenal varices (DVs) are ectopic gastrointestinal varices (ECVs) associated with portal hypertension (PH). We present the case of an 82‐year‐old woman who presented with symptomatic anemia secondary to DV hemorrhage diagnosed on oesophagogastroduodenoscopy. This lesion was treated with endoscopic adrenaline injection and clip application. The patient re‐presented on multiple occasions with bleeding recurrence localized to the duodenum, which was managed with intramuscular octreotide and oral beta‐blockade resulting in sustained remission of bleeding. This case highlights a rare cause of upper gastrointestinal hemorrhage and highlights the value of somatostatin analogues for conservative treatment of DVs.

## Graphical abstract

Duodenal varices are ectopic gastrointestinal varices associated with portal hypertension. This case highlights a rare cause of upper gastrointestinal hemorrhage in the absence of portal hypertension and highlights the value of somatostatin analogs for conservative treatment of duodenal varices.

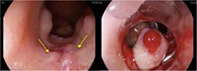

## Introduction

Ectopic varices (ECV) are pressurized portosystemic collaterals that occur throughout the gastrointestinal tract, accounting for 5% of all variceal bleeds, 20% of which are duodenal in origin.^1^ The majority of duodenal varices (DVs) arise at the duodenal bulb, most commonly due to portal hypertension (PH) attributable to hepatic cirrhosis.^2^ DV hemorrhage is rare and difficult to manage, with high recurrence and mortality rates.^3^

Management strategies for DVs currently focus on endoscopic, radiological, surgical, and medical modalities; however, there is no consensus on the most appropriate therapy.

We present the case of an 82‐year‐old woman who presented to our institution with symptomatic anemia and melena who was diagnosed with an upper gastrointestinal (UGI) hemorrhage secondary to a DV in the context of previous abdominal surgery.

## Case summary

An 82‐year‐old female was brought in by ambulance to a metropolitan emergency department with fatigue and a hemoglobin (Hb) of 70 g/L. Past medical history was significant for breast cancer requiring right mastectomy; ovarian cancer requiring oophorectomy and adjuvant chemotherapy 25 years prior; subsequent metastatic recurrence requiring distal pancreatectomy, splenectomy and cholecystectomy; small bowel obstruction managed non‐operatively; appendicectomy, hypertension, and type two diabetes mellitus. Five months prior to presentation the patient had undergone oesophagogastroduodenoscopy (OGD) with endoscopic ultrasound for assessment of abdominal pain, liver function test (LFT) derangement, and common bile duct dilation, which was normal.

Her regular medications were amlodipine 5 mg daily, doxepin 25 mg nocte, gliclazide SR 60 mg daily, metformin 500 mg twice daily, esomeprazole 20 mg once daily, vildagliptin 50 mg once daily, and budesonide/formoterol inhaler 100 mcg/3 mcg one puff twice daily.

Clinical examination was notable for melaena on rectal examination. A previous laparotomy scar was present. There were no peripheral signs suggestive of chronic liver disease. Biochemical assessment demonstrated a Hb of 67 g/L, urea of 9.3 μmol/L, Ferritin of 29 μg/L. She was transfused four units of cross‐matched blood, and an iron infusion and pantoprazole infusion were commenced. Inpatient gastroscopy was notable for a single nonbleeding erosion in the middle third of the esophagus, while colonoscopy was noncontributory. Capsule endoscopy on discharge was normal.

Two months later the patient re‐presented with a 3‐day history of melaena and Hb of 67 g/L. Gastroscopy on day 2 of admission showed an abnormal vascular structure suggestive of a varix in the first portion of the duodenum, which extended semi‐circumferentially on the posterior inferior wall of the duodenum. This bled on contact requiring adrenaline injection and hemospray for hemostasis.

Computed tomography mesenteric angiogram (CTMA) was performed, which demonstrated multiple mucosal prominent tortuous vascular structures suggestive of varices at D1 and the duodenal bulb. There was no active arterial hemorrhage amenable to embolization.

**Figure 1:**
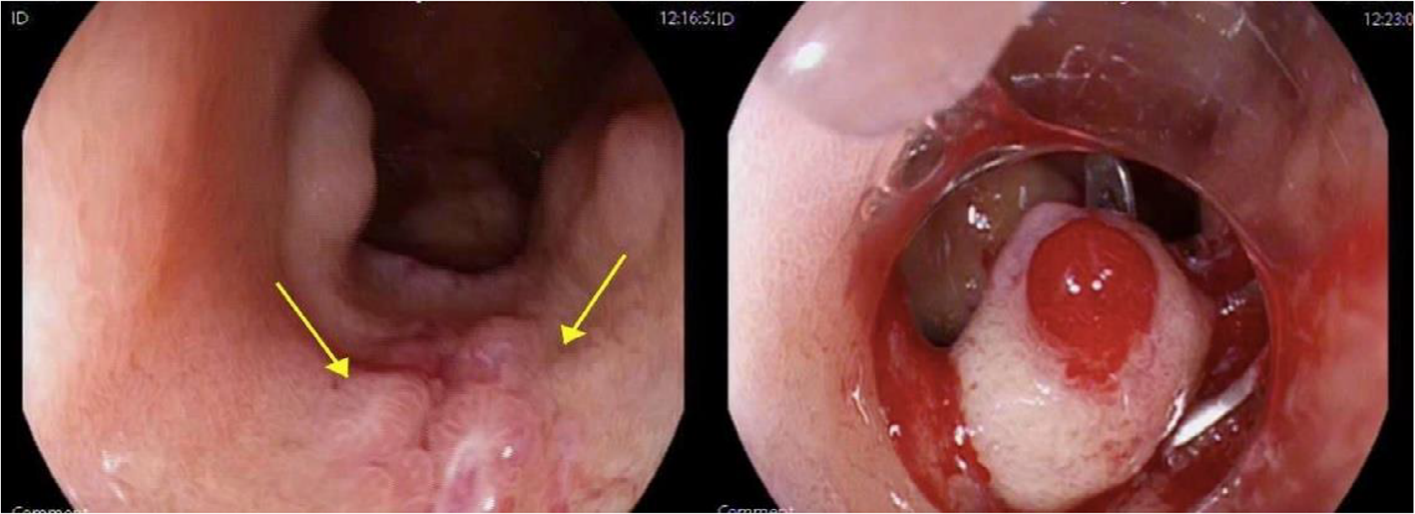
Oesophagogastroduodenoscopy demonstrating a prominent submucosal vessel at D1; OVESCO over the scope clip applied.

Repeat gastroscopy 24 h later demonstrated a prominent submucosal structure in D1 with a vessel visible in the submucosa (Fig. 1). An OVESCO over the scope clip was applied to the area of concern.

CTMA also demonstrated significantly distorted anatomy secondary to her previous intra‐abdominal surgery. A transhepatic portogram was proposed but later abandoned as the abnormal vasculature was circumferential around the duodenum with multifocal points for potential bleeding. An interventional radiologically guided approach for embolization was deemed to be high risk due to the potential for inadvertent rupture of adjacent collateral venous vessels. Surgical opinion was sought for definitive management; however, due to significant comorbidities, it was deemed not feasible. A conservative approach consisting of prophylactic carvedilol 12.5 mg BD and transfusion support was pursued.

A red cell scan following another episode of bleeding and negative gastroscopy showed a small moderate focus of uptake in the medial wall of D1. This was not actively bleeding and corresponded with the endoscopically visualized vascular lesions.

To reduce portal pressures, intramuscular octreotide 10 mg every 4 weeks was commenced. At the time of manuscript submission, the patient had not presented for 4 months after commencement of IM octreotide and carvedilol therapy.

## Discussion

DVs contribute to 17% of all ECV bleeding and are observed in up to 40% of patients diagnosed with PH; however, they are not usually associated with bleeding in this population.^1^, ^4^ Mortality associated with DV hemorrhage is high and a lack of experience secondary to its relatively low incidence makes diagnosis and treatment difficult.

DVs are associated with PH, with liver cirrhosis being the most common pathology implicated.^5^ With regards to extrahepatic causes of DVs, occlusion of the splenic or portal vein make up 50% of diagnosed cases.^6^ It has also been postulated that DVs may develop after endoscopic band ligation (EBL) secondary to spontaneous portosystemic shunting, highlighting a treatment dilemma.

There is minimal literature focusing on the management of DV hemorrhage due to the infrequency of the event. Initial treatment focuses on resuscitation, somatostatin analogue therapy, and vasopressor support as required.^7^

EBL has previously been utilized; however, it is limited by the field of view endoscopically, along with a high rate of rebleeding and recurrence.^8^ Endoscopic injection of cyanoacrylate (or endoscopic injection sclerotherapy [EIS]) has demonstrated some efficacy in observational studies. High postsurgical mortality rates have resulted in surgical procedures being superseded by endoscopic and radiological alternatives, which have demonstrated modest success.^9^ Medical management of EVs is lacking; however, somatostatin analogue therapy has proved to have merit in preventing intestinal variceal bleeding.^10^

This is a case of DV secondary to scar tissue that was still amenable to treatment with beta‐blockade and octreotide. On further assessment, no pre‐hepatic, posthepatic, or intrahepatic cause was identifiable that would constitute a diagnosis of noncirrhotic PH. It is postulated that previous abdominal surgery with resection of venous vasculature resulted in the formation of collateral vessels around the duodenum, subsequently resulting in hemorrhage.

This case highlights an uncommon presentation of UGI hemorrhage and highlights the dilemmas associated with the management of DVs. Management with beta‐blockade and somatostatin analogue therapy was beneficial and should be considered in refractory bleeding cases.

*Informed consent statement*: Written informed consent was obtained from the presented patient prior to publication.

## References

[1] Norton ID , Andrews JC , Kamath PS . Management of ectopic varices. Hepatology. 1998; 28: 1154–8. 9755256 10.1002/hep.510280434

[2] Yipeng W , Anjiang W , Bimin L , Chenkai H , Size W , Xuan Z . Clinical characteristics and efficacy of endoscopic treatment of gastrointestinal ectopic varices: a single‐center study. Saudi J. Gastroenterol. 2021; 27: 35–43. 33208560 10.4103/sjg.SJG_50_20 PMC8083249

[3] Henry Z , Uppal D , Saad W , Caldwell S . Gastric and ectopic varices. Clin. Liver Dis. 2014; 18: 371–88. 24679501 10.1016/j.cld.2014.01.002

[4] Al‐Mofarreh M , Al‐Moagel‐Alfarag M , Ashoor T , Shadoochy F . Duodenal varices. Report of 13 cases. Zeitschrift fur Gastroenterologie. 1986; 24: 673–80. 3101297

[5] Saad WE , Lippert A , Saad NE , Caldwell S . Ectopic varices: anatomical classification, hemodynamic classification, and hemodynamic‐based management. Tech. Vasc. Interv. Radiol. 2013; 16: 158–75. 23830673 10.1053/j.tvir.2013.02.004

[6] Kotfila R , Trudeau W . Extraesophageal varices. Dig. Dis. 1998; 16: 232–41. 9732183 10.1159/000016871

[7] Akhter NM , Haskal ZJ . Diagnosis and management of ectopic varices. Gastrointest. Interv. 2012; 1: 3–10.

[8] Shiraishi M , Hiroyasu S , Higa T , Oshiro S , Muto Y . Successful management of ruptured duodenal varices by means of endoscopic variceal ligation: report of a case. Gastrointest. Endosc. 1999; 49: 255–7. 9925710 10.1016/s0016-5107(99)70498-0

[9] Sato T , Akaike J , Toyota J , Karino Y , Ohmura T . Clinicopathological features and treatment of ectopic varices with portal hypertension. Int. J. Hepatol. 2011; 2011: 1–9. 10.4061/2011/960720 PMC3170857 21994879

[10] Dray X , Vahedi K , Odinot J‐M , Marteau P . Octreotide for recurrent intestinal variceal bleeding in patients without portal hypertension. Eur. J. Gastroenterol. Hepatol. 2009; 21: 836–9. 19381096 10.1097/MEG.0b013e328310abd1

